# Attentional Bias for Uncertain Cues of Shock in Human Fear Conditioning: Evidence for Attentional Learning Theory

**DOI:** 10.3389/fnhum.2017.00266

**Published:** 2017-05-23

**Authors:** Stephan Koenig, Metin Uengoer, Harald Lachnit

**Affiliations:** Department of Psychology, Philipps-Universität MarburgMarburg, Germany

**Keywords:** associative learning, fear conditioning, uncertainty, attention, eye movements

## Abstract

We conducted a human fear conditioning experiment in which three different color cues were followed by an aversive electric shock on 0, 50, and 100% of the trials, and thus induced low (L), partial (P), and high (H) shock expectancy, respectively. The cues differed with respect to the *strength of their shock association* (L < P < H) and the *uncertainty* of their prediction (L < P > H). During conditioning we measured pupil dilation and ocular fixations to index differences in the attentional processing of the cues. After conditioning, the shock-associated colors were introduced as irrelevant distracters during visual search for a shape target while shocks were no longer administered and we analyzed the cues’ potential to capture and hold overt attention automatically. Our findings suggest that fear conditioning creates an automatic attention bias for the conditioned cues that depends on their correlation with the aversive outcome. This bias was exclusively linked to the strength of the cues’ shock association for the early attentional processing of cues in the visual periphery, but additionally was influenced by the uncertainty of the shock prediction after participants fixated on the cues. These findings are in accord with attentional learning theories that formalize how associative learning shapes automatic attention.

## Introduction

For a long time, attention research has focused on the conceptual dichotomy of bottom-up (exogenous) versus top-down (endogenous) processing (Wolfe et al., 1989; Desimone and Duncan, 1995; Itti and Koch, 2001; Corbetta and Shulman, 2002). From this perspective, stimuli in our sensory environment attract attention because they are either of high physical salience, or their selection is relevant for performing a task (goal-directed, strategic selection). However, this conceptual dichotomy fails to account for converging empirical evidence of automatic attentional capture by non-salient and task-irrelevant stimuli that previously acquired *value* by association with reward or punishment (Awh et al., 2012; Anderson, 2015; Le Pelley et al., 2016). For example, in several recent studies (Anderson et al., 2011a,b; Anderson and Yantis, 2012; Le Pelley et al., 2015), a specific color was repeatedly paired with monetary gain to establish an association between that color and reward. Following this training, the same color was introduced as a task-irrelevant distracter during search for a shape target, while reward was no longer available. With this design, reward-associated color distracters captured attention automatically and strongly interfered with finding the shape target.

It has been demonstrated that this automatic bias, does not exclusively result from pairings with reward in appetitive conditioning, but also results from aversive conditioning in which stimuli become associated with monetary loss, electric shock, or loud noise (Wang et al., 2013; Wentura et al., 2014; Schmidt et al., 2015). In a recent series of experiments Wang et al. (2013) examined the effects of appetitive and aversive learning on value-based attentional capture. They demonstrated that both, distracters associated with monetary gain *or* monetary loss, elicited stronger attentional capture than distracters that were associatively neutral (Experiment 1). Additionally, an association with monetary loss was even more effective than monetary gain to induce value-based capture for physically weak distracters with little bottom-up salience in comparison to the target (Experiment 2). The authors also examined attentional capture of distracters that were previously used as conditioned stimuli (CS) in human fear conditioning (Experiment 3). They reported that a distracter previously followed by an aversive electric stimulation on 75% of the conditioning trials (CS+) induced stronger value-based capture than a distracter that had never been followed by shock (CS-).

**FIGURE 1:**
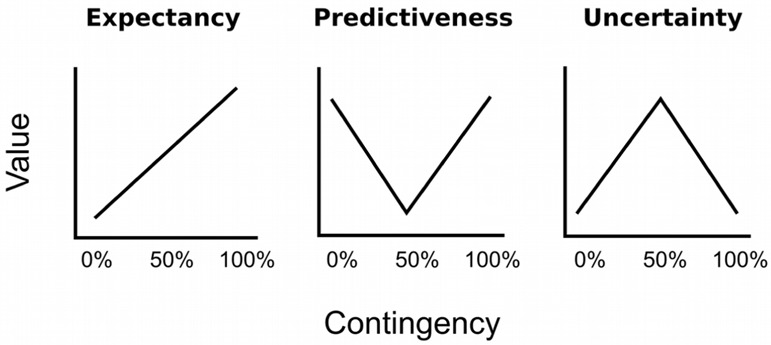
**Hypothetical experiment in which the conditioned stimulus (CS; cue) is followed by the unconditioned stimulus (US; outcome) on 0, 50, or 100% of the trials.** The 0% cue reliably predicts the absence of the US, the 100% cue reliable predicts the occurrence of the US, the 50% induces uncertainty, and consistently causes error in the prediction of the US. From left to right, the panels depict different perspectives on how associative learning ascribes value to the CS based on these contingency. This learned value in turn might create an attention bias that influences automatic attentional capture by the cues. The **Left** panel depicts the idea that attention linearly increases with the strength of the association between the CS and the US. In the **Mid** panel attention is selectively allocated to cues that are good predictors of the outcome. The **Right** panel depicts the idea that learners allocate attention to uncertain cues. Figure adopted from Gottlieb (2012).

Experimental demonstrations of value-based attentional capture have mainly focused on *value* as the strength of the learned association between the stimulus (color) and the significant outcome (reward, punishment): The stronger this association, the higher the value, and the stronger the potential of a stimulus to capture attention (for review see Le Pelley et al., 2016). For example, a distracter previously associated with a large reward has been reported to exhibit a higher probability to capture attention than a distracter previously associated with a small reward (Anderson et al., 2011b; Anderson and Yantis, 2013). Likewise, a stimulus followed by electric shock on 75 or 100% of the trials exhibited stronger capture than a stimulus that was never followed by shock during conditioning (Wang et al., 2013; Schmidt et al., 2015). The idea that attention to a stimulus should increase with the strength of its association to a significant outcome is depicted in the left panel of **Figure 1**. If three different cues were followed by electric shock on 0, 50, or 100% of the trials, respectively, the 100% cue should become more strongly associated with shock than the 50% cue, which in turn should hold a stronger association than the 0% cue. This rank order is predicted by most quantitative models of error-driven associative learning like the model of Rescorla and Wagner (1972). If attention is driven by the strength of such an association, the attention bias should exhibit the same linear increase with outcome contingency. An alternative perspective on how learning history creates attentional bias has been provided by associative learning theories which assume that not associative strength *per se* but rather the *prediction error* is critical for creating an attention bias (Mackintosh, 1975; Pearce and Hall, 1980). This error represents the discrepancy between the predicted outcome and the actual outcome. For example, after participants have learned the outcome contingencies of 0, 50, or 100% from our example, they will be able to fully anticipate the omission or occurrence of shock after the 0% cue or the 100% cue, respectively, and these cues thus repeatedly occur with no prediction error. In contrast, the outcome cannot be anticipated for the uncertain 50% cue, which in turn consistently occurs with a high prediction error. The mid and right panel of **Figure 1** depict two antagonistic perspectives on how this higher prediction error for the 50% cue creates attention bias. According to Mackintosh (1975), participants selectively attend to stimuli that are predictive of their consequence and thus exhibit a low prediction error. As shown in the mid panel of **Figure 1**, cues reinforced at a rate of 0 and 100% thus should exhibit a higher attention bias than an uncertain 50% cue, because they perfectly predict the omission or occurrence of reward, respectively. In contrast, as shown in the right panel of **Figure 1**, the learning theory of Pearce and Hall (1980; Pearce et al., 1982) posits that learners acquire a bias to attend to uncertain cues which consistently occur in the presence of a high prediction error. From this perspective, attention to uncertain cues must be upheld in order to keep it accessible to further learning that might improve the prediction in the future.

There is physiological evidence from single cell recordings in the monkey that the dopaminergic midbrain computes values of both, expectancy (associative strength) and uncertainty (prediction error) in order to engage in learning and decision making (Fiorillo et al., 2003; Schultz et al., 2008). In human fear conditioning, there is fMRI evidence for two distinct patterns of activity in disparate brain regions. For example, Dunsmoor et al. (2007) reported that activity in amygdala, anterior cingulate, and fusiform gyrus linearly increased with shock expectancy, whereas insula and dorsolateral prefrontal cortex showed highest activity to uncertain cues. The concurrent representation of expectancy and uncertainty also is supported by behavioral evidence. Kaye and Pearce (1984) reported that during appetitive conditioning with rats the conditioned response (magazine approach) elicited by a light cue was linked to the expectancy of food, while the orienting response (rearing to the light) was linked to the lights uncertainty in predicting food. For human appetitive learning, Koenig et al. (unpublished) found that both values affected overt attention, where expectancy was linked to attentional capture (capture frequency), and uncertainty was linked to attentional holding (capture duration).

In the current experiment we examined whether the correspondence of attentional capture and attentional holding to expectancy and uncertainty we previously reported for appetitive learning also holds for learning about an aversive outcome. In the first stage of the experiment we used a fear conditioning procedure in which three different color cues were followed by an aversive electric shock in 0, 50, or 100% of the trials, respectively. In the second stage of the experiment, previously trained colors were introduced as task-irrelevant color distracters during visual search for a shape singleton. Both, the conditioning procedure and the subsequent search task were designed to allow for the measurement of ocular fixations. We analyzed fixation frequency and fixation latency as measures of attentional capture, and fixation duration as a measure of attentional holding. During fear conditioning, we also recorded pupil size, as a proxy of emotional arousal (Bradley et al., 2008). Pupil size has been previously used to index conditioned fear (Reinhard and Lachnit, 2002; Reinhard et al., 2006), but has also been linked to attentional orienting (Sokolov, 1963; Lynn, 1966; Geva et al., 2013; Corneil and Munoz, 2014; Wang and Munoz, 2015) and the processing of uncertainty (Richer and Beatty, 1987; Satterthwaite et al., 2007; Jepma and Nieuwenhuis, 2011; Preuschoff et al., 2011; Koenig et al., unpublished). Based on these prior findings, we expected pupil size to be affected by both shock expectancy and shock uncertainty.

## Materials and Methods

The study was approved by the local ethics committee of the Faculty of Psychology at the Philipps-Universität Marburg (AZ: 2012-07, 2013-28k). All subjects gave written informed consent in accordance with the Declaration of Helsinki.

### Participants

Thirty-two students of the Philipps-Universität Marburg participated in the experiment and received either course credit or payment. All participants had normal or corrected-to-normal vision. Twenty-five of the participants were female and seven were male. Their age ranged from 19 to 34 years, (*M* = 23.09, *SD* = 3.753).

### Apparatus

Testing took place in a sound-attenuated, dimmed room. Monocular eye movements were recorded using an infrared video-based eye tracker (Eyelink 2000, SR-Research, Mississauga, ON, Canada) that sampled gaze position and pupil size at a frequency of 1000 Hz. Sampling of the left versus right eye was counterbalanced across participants. The eye tracker restrained the participants head via chin and forehead rests and was table-mounted in front of a 22″-CRT monitor (Iiyama, Vision Master Pro514) yielding an eye-to-screen-distance of 78 cm. The eye tracker was calibrated with a 9-point grid of calibration targets. For each participant, the calibration procedure was repeated until subsequent validation confirmed a maximal calibration error <0.5°.

Presentation of all stimuli was controlled by Presentation^®^ software^1^. A 10 ms dc electric shock was used as the aversive unconditioned stimulus and was delivered via silver–silver chloride electrodes to the volar surface of the participant’s right arm from an isolated transformer–condenser shock generator (Kimmel et al., 1980).

### Stimuli

**FIGURE 2:**
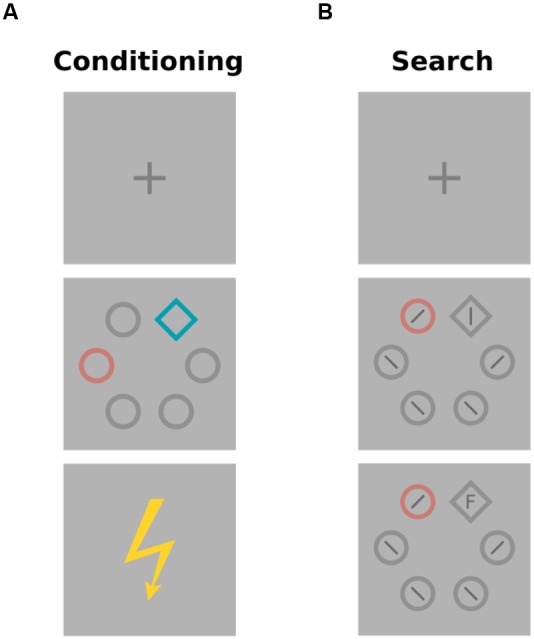
**All trials started with a 2-s fixation cross. (A)** In conditioning trials the subsequent search array was presented for 5 s and included a colored annulus and a colored diamond amongst gray distracter stimuli. Participants were instructed to attend to the color of the annulus in order to predict whether an electric shock was going to be delivered subsequently. **(B)** In search trials, previously trained colors were introduced as distracters to interfere with the required eye movement to a shape singleton (diamond).

**FIGURE 3:**
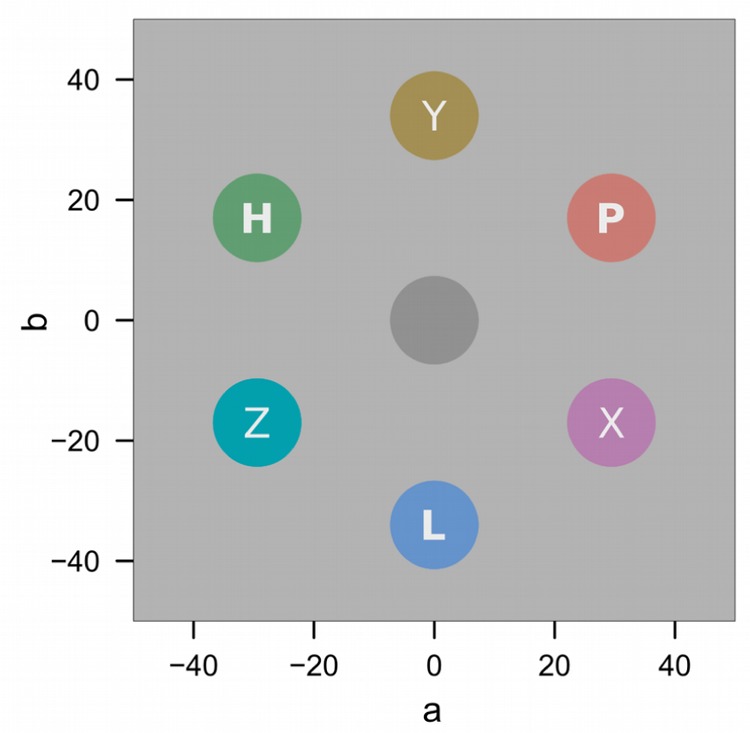
**Stimulus colors in CIE L^∗^a^∗^b space.** Letters L, P, H illustrate assignment of colors to the low, partial, and high expectancy conditions. Letters X, Y, Z illustrate assignment of colors to the diamond distracters.

**Figure 2A** depicts the sequence of events during conditioning trials. All stimuli were presented on a light gray (*L*^∗^ = 87) background. After a 2-s fixation cross, a colored annulus (31 mm) and a colored diamond (35 mm) were presented amongst four gray annuli (31 mm) for 5 s. The six stimuli were evenly spaced on an imaginary circle, placed at a distance of 100 mm to the center of the computer screen (7.34 degrees of visual angle; dva), and rendered with a line width of 4 mm. The colored annulus was the relevant conditioned stimulus (CS), and in each trial was rendered in one of three different colors (for example red, green, or blue). The colored diamond was a distracter stimulus, and in each trial was rendered in one of three different colors (e.g., cyan, magenta, yellow). The CS and distracter color sets were constructed from six colors equidistant in CIE L^∗^a^∗^b color space as shown in **Figure 3**. Across both sets, colors were matched for lightness (*L*^∗^ = 60) and chroma (*C*^∗^ = 34). Hues in the first set were *h* = 30 (red), 150 (green), and 270 (blue), respectively. Hues in the second set were h°= 90 (yellow), 150 (cyan), and 270 (magenta). For one half of the participants CS colors were drawn from the first color set, and distracter colors were drawn from the second set, while the reverse was true for the other half of the participants. Also, the assignment of the CS colors to experimental conditions of low (L), partial (P), or high (H) shock expectancy (with 0, 50, or 100% shock contingency, respectively) was counterbalanced across participants and **Figure 3** only illustrates one concrete example.

**Figure 2B** depicts the stimuli presented in search trials. A small vertical or horizontal line was presented inside the gray diamond target, while oblique lines were presented inside the distracter annuli. In 75% of the trials one distracter annulus was rendered in one of the CS colors from the conditioning stage.

### Procedure

On arrival, written instructions were presented, and participants gave informed consent to the aversive conditioning procedure and the subsequent search task. Before the experiment started, the experimenter attached the shock electrodes to the participants arm and delivered shocks of increasing intensity for participants to choose an intensity that was “definitely unpleasant but not really painful.” Participants were assured that this intensity would not change during training. The eye tracker then was calibrated as described above, and the experiment started with 45 trials of aversive conditioning that were followed by 240 search trials

#### Aversive Conditioning

All trials began with a 2-s fixation cross that instructed participants to stop blinking and pay attention (**Figure 2A**). The subsequent search display presented the colored annulus CS and the diamond distracter. In reinforced trials, the electric shock was presented at the time the stimulus display disappeared. The electric shock never occurred after one CS color (0% expectancy), occurred at random after a second CS color (50% expectancy), and always occurred after the third CS color (100% expectancy). These contingencies established trained colors as low expectancy cues (L), partially rewarded cues (P), and high expectancy cues (H), respectively. Over the course of conditioning, the three CS colors occurred in the context of each of the three distracter colors, rendering the distracter irrelevant for the prediction of the impending shock. In abstract notation, participants received training, LX-, LY-, LZ-, PX±, PY±, PZ±, HX+, HY+, HZ+, where L, P, and H denote relevant CS colors, X, Y, and Z denote irrelevant distracter colors, and -, ±, and + denote the application of shock in 0, 50, or 100% of the trials, respectively. Within each of three consecutive blocks of conditioning trials, each CS color (L, P, and H) was presented six times, resulting in 54 conditioning trials in total, with 18 presentations of each color. Trials were presented in a different pseudo-random sequence for each participant, with the restriction that electric shock would not exceed three presentations in a row. Written instructions for the conditioning stage were as follows: “Please attend to the color of the annulus. This color may provide information about whether you are to experience an electrical stimulation at the end of the trial. The color of the diamond is irrelevant.” Participants thus were pointed to the fact that the annulus color was the relevant information in the display, but they were not instructed about the exact contingencies of the CS colors which rather had to be learned during conditioning.

#### Visual Search Task

After conditioning, participants performed 240 search trials. All trials began with a 2-s fixation cross. In the subsequent search display participants were required to look at the shape target as quickly as possible, and identify the line orientation in the target by pressing a right versus left mouse button. The search display could contain only gray distracters in the baseline condition, or could feature one annulus that was rendered in one of the previously trained annulus colors L, P, or H. Search trials were administered in five consecutive blocks of 48 trials. Each block featured 12 baseline trials with no color distracter, and 36 trials featuring a colored distracter (with 12 trials per trained color). Within each block of search trials, the diamond target appeared at all six positions of the search array an equal number of times. The color distracter always was presented at one of the neighboring positions. Within each search block, participants received a different pseudo-random sequence of trials, with the restriction, that the same distractor color did not occur more than three times in a row. Participants were instructed that there would be no further shocks in the search task, that they had to respond to the shape target as quickly as possible, and that “all circles in the task could be ignored.”

### Dependent Variables and Data Analysis

Custom MATLAB (The MathWorks, Inc., 2012) software was used for the signal conditioning of gaze position traces and pupil size, and the velocity-based detection (threshold 30°/s) and parametrization ocular fixations (Koenig, 2010; Koenig and Lachnit, 2011).

#### Pupil Size

In conditioning trials, we recorded changes in pupil size as a measure of autonomic arousal elicited by the fear-conditioned color stimuli in anticipation of the omission or occurrence of electric shock. Pupil traces were normalized with reference to the initial pupil size at the time the search array appeared. The normalized traces were computed as the change in pupil size as the percentage of initial size. To analyze how differential responding to the CS developed within trial, the 5-s CS interval was partitioned into four successive bins of 1250 ms for statistical analysis (Reinhard et al., 2006).

#### Total Dwell Time

During the CS interval participants were allowed to move their eyes in order to explore the search display and fixate on the colored annulus (relevant CS), the diamond shape (irrelevant distracter), or any of the other circular gray distracters. We summed the duration of fixations on the colored annulus within trial to compute the total dwell time on the relevant CS. Previous studies have used such total dwell as measures of overt attention during associative learning (Hogarth et al., 2008; Le Pelley et al., 2011).

#### Capture Frequency

In learning trials, the shock-associated color was always presented in the context of an irrelevant shape distracter of another color. We analyzed the frequency of trials in which the irrelevant shape distracted from the relevant color cue and captured the first fixation. In search trials, participants had to find the shape target as quickly as possible while a simultaneous color distracter previously associated with the omission or presentation of shock was now irrelevant and could have been ignored. We were interested in the potential of the color distracters to automatically capture attention, and analyzed the frequency of trials, in which participants erroneously fixated on the color distracter before fixating the shape target.

#### Capture Duration

If participants erroneously fixated the color distracter in search trials, we analyzed the dwell time on the distracter before participants eventually moved their eyes to the shape target.

All statistical analyses were conducted using the R language and environment for statistical computing (R Core Team, 2016). Analysis of variance (ANOVA) and contrast analysis was used for all dependent variables except capture duration. For all ANOVAs, reported p-values are adjusted according to Huynh and Feldt (1976). Degrees of freedoms are stated uncorrected. Capture duration could only be computed from an unbalanced subset of trials (capture trials), and effects were estimated using a linear-mixed model (LMM; Bates et al., 2015). The model included a maximal random effect structure as recommended by Barr et al. (2013). Approximate degrees of freedom for *F*- and *t*-statistics were computed using the method of Kenward and Roger (1997; Halekoh and Højsgaard, 2014).

We used contrasts as a focused test of the influence of expectancy and uncertainty on attention. Contrast were computed from the ANOVA’s /LMM’s prediction of marginal means (Goodnight and Harvey, 1997; Lenth, 2016). With reference to theoretical predictions in **Figure 1**, a linear contrast with coefficients -1, 0, 1, coded for the hypothesis that attention should increase with shock expectancy. A quadratic contrast, -0.5, 1, -0.5, coded for the hypothesis of attention to uncertain cues. A third contrast, with coefficients -0.75, 0.50, 0.25, explored the hypothesis of a combined influence of both, expectancy and uncertainty. To account for simultaneous inference in the context of non-orthogonality, contrasts tests were adjusted according to Westfall (1997; Hothorn et al., 2008).

## Results

### Fear Conditioning

#### Pupil Size

**FIGURE 4:**
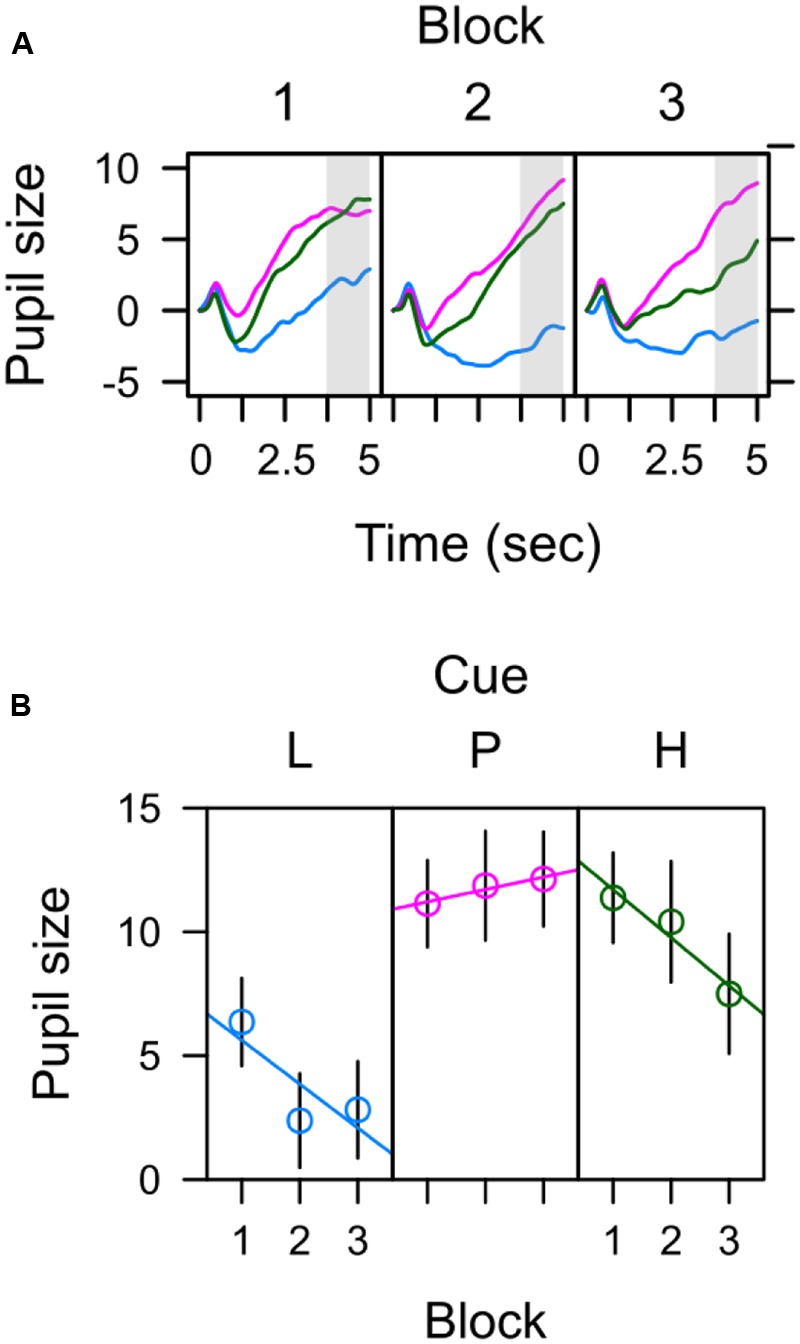
**Pupil size elicited by low (blue), partial (red), and high (green) expectancy of electric shock. (A)** Average pupil traces in the 5-s CS interval **(B)** Pre-US pupil size during the last 1250 ms preceding the US (3750–5000 ms).

**Figure 4A** depicts average pupil traces elicited by the low expectancy cue (L), the partially reinforced cue (P), and the high expectancy cue (H). For statistical analysis the trial sequence was partitioned into three successive blocks of training. Within trial, the 5-s CS interval was partitioned into four successive bins of 1250 ms. An ANOVA with factors CS, block, and bin revealed no effect of block, *F* < 1, but significant main effects of CS, *F*(2,62) = 6.687, *p* = 0.004, $\eta_{p}^{2}$ = 0.177, and bin, *F*(3,93) = 31.620, *p* < 0.001, $\eta_{p}^{2}$ = 0.505, that were modulated by a CS × Bin interaction, *F*(6,186) = 7.347, *p* < 0.001, $\eta_{p}^{2}$ = 0.192. Simple effects of CS in each successive interval are shown in **Table 1** and reveal that differences in pupil size gradually increased to reach a maximum in the last interval (highlighted gray in **Figure 4A**). For this last interval directly preceding the US, contrasts in the top row of **Table 2** make clear that the pattern of data best fitted with a combined influenced of expectancy and uncertainty on pupil dilation. Pairwise comparison in **Table 3** indicated stronger dilation for the high expectancy and the partial cue than for the low expectancy cue. Taken together, our analysis suggests that participants encoded values of both, the strength of the shock associations and the uncertainty of predicting shock (prediction error). The assumed integration of both values is illustrated in **Figure 7**.

**Table 1:** Simple effects of CS in four successive time windows during the 5 s CS interval.

Time window	*F*(2,62)	$\eta_{p}^{2}$	*p*
0–1250 ms	1.292	0.040	0.281
1250–2500 ms	4.579	0.129	0.017*
2500–3750 ms	7.468	0.194	0.003**
3750–5000 ms	7.695	0.199	0.002**

*N = 32; ^∗^*p* < 0.05, ^∗∗^*p* < 0.01, and ^∗∗∗^*p* < 0.001.*

**Table 2:** Contrasts comparing low expectancy (L), partial reinforcement (P), and high expectancy (H).

		Expectancy	Uncertainty	Combined
**Stage 1: Fear conditioning**				
*Pupil dilation*	t(62)	2.832	2.715	3.918
	*p*	0.003**	0.004^∗∗^	0.001***
	β	0.398	0.382	**0.551**
*Dwell time*	t(62)	4.000	2.205	4.468
	*p*	0.001***	0.016*	0.001***
	β	0.232	0.128	**0.260**
*Target fixation latency*	t(108)	-2.657	-0.639	-2.427
	*p*	0.011*	0.262	0.011*
	β	**-0.313**	-0.075	-0.286
*Distracter fixation latency*	t(108)	2.596	0.130	2.048
	*p*	0.013*	0.448	0.021*
	β	**0.306**	0.015	0.241
*Capture frequency (by shape)*	t(62)	-3.230	-0.203	-2.575
	*p*	0.003**	0.420	0.006**
	β	**-0.687**	-0.043	-0.548
**Stage 2: Visual search task**				
*Capture frequency (by color)*	t(62)	3.172	0.195	2.525
	*p*	0.003**	0.423	0.007**
	β	**0.249**	0.015	0.200
*Capture duration*	t(21)	1.573	2.210	2.665
	*p*	0.065	0.019*	0.017*
	β	0.142	0.203	**0.241**

*The expectancy contrast codes for a linear increase L < P < H with coefficients -1, 0, 1. The uncertainty contrast codes for the pattern L < P > H with coefficients -0.5, 1, -0.5. The third contrast explores the possibility that attention is influenced by a combination of both values and has coefficients -0.75, 0.50, 0.25. Tests are one-sided, to test for the specified patterns (and not for the inverse patterns); with ^∗^*p* < 0.05, ^∗∗^*p* < 0.01, and ^∗∗∗^*p* < 0.001. Effect sizes are computed for standardized variables (strongest effect highlighted in bold).*

**Table 3:** Pairwise comparison between low expectancy (L), partial reinforcement (P), and high expectancy (H) cues.

		L-P	L-H	P-H
**Stage 1: Fear conditioning**				
*Pupil dilation*	t(62) *p* β	-3.767 0.001^∗∗∗^ -0.530	-2.832 0.003^∗∗^ -0.399	0.936 0.177 0.132
*Dwell time*	t(62) *p* β	-3.910 0.001^∗∗∗^ -0.227	-0.400 0.001^∗∗∗^ -0.232	-0.090 0.536 -0.005
*Target fixation latency*	t(108) *p* β	1.882 0.031^∗^ 0.222	2.657 0.005^∗∗^ 0.313	0.775 0.219 0.091
*Distracter fixation latency*	t(108) *p* β	-1.411 0.081 -0.166	-2.596 0.005^∗∗^ -0.306	-1.186 0.119 -0.139
*Capture frequency (by shape)*	t(62) *p* β	1.791 0.039^∗^ 0.381	3.230 0.001^∗∗∗^ 0.687	1.439 0.077 0.306
**Stage 2: Visual search task**				
*Capture frequency*	t(62) *p* β	-1.755 0.042^∗^ -0.138	-3.172 0.001^∗∗∗^ -0.249	-1.418 0.081 -0.112
*Capture duration (by color)*	t(21) *p* β	-2.720 0.006^∗∗^ -0.247	-1.573 0.065 -0.142	1.137 0.134 0.104

*Tests are one-sided and refer to the difference between means that is predicted by the contrast with the highest effect size in **Table 2**; ^∗^*p* < 0.05, ^∗∗^*p* < 0.01, and ^∗∗∗^*p* < 0.001. For example, tests for pupil dilation are L < P, L < H, and P > H.*

#### Total Dwell Time on CS

**FIGURE 5:**
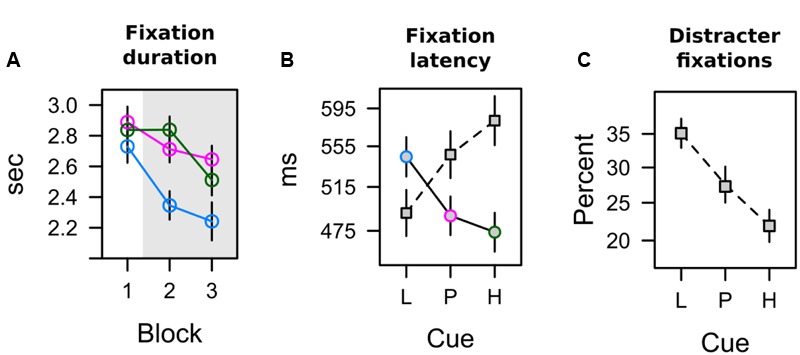
**Overt attention in the learning task (A)** Duration of fixations on cues with low [blue], partial [red], and high [green] shock expectancy. **(B)** Latency of the first fixation on the circular color cue [solid line] and the diamond distracter [dashed line]. **(C)** The percentage of distracter fixations depends upon how strongly the color cue is associated with shock.

The total dwell time on the CS during conditioning is shown in **Figure 5A**. A 3 × 3 ANOVA with factors CS (L, P, H) and block (1, 2, 3) revealed a significant effect of CS, *F*(2,62) = 11.458, *p* < 0.001, $\eta_{p}^{2}$ = 0.270, a main effect of block that fell short of significance, *F*(2,62) = 2.879, *p* = 0.083, $\eta_{p}^{2}$ = 0.085, and no interaction, *F*(4,124) = 1.899, *p* = 0.126, $\eta_{p}^{2}$ = 0.058. Simple effect analysis revealed no differences in total dwell time in the first block of conditioning, *F*(2,62) = 1.621, *p* = 0.209, $\eta_{p}^{2}$ = 0.050, but a significant effect in the second block, *F*(2,62) = 6.793, *p* = 0.003, $\eta_{p}^{2}$ = 0.180, and third block, *F*(2,62) = 7.379, *p* = 0.003, $\eta_{p}^{2}$ = 0.192. For these last two blocks of training (highlighted gray in **Figure 5A**), the pattern of data was in accord with a combined influence of expectancy and uncertainty as depicted in the second row of **Table 2**. Pairwise comparison in **Table 3** indicated that in comparison to the low expectancy cue dwell times were longer on the partially reinforced cue and the high expectancy cue which did not differ from each other. Please note that the absence of this difference was not due to a ceiling effect: Inspection of **Figure 5A** makes clear that total dwell time did not exceed 3 s even in the first block of training, and that dwell time decreased in the course of further training. In contrast, the stimuli were shown for a total of 5 s, and our design thus would have allowed for more prolonged dwell time on the partial or high expectancy cue in principle. The observed pattern L < P = H best fitted with a joint influence of both associative strength and uncertainty.

#### CS Fixation Latency

Both measures reported above, pupil size and total dwell time, were influenced by late processing in the 5-s interval of stimulus presentation. Differences in elicited pupil dilation were maximal in the *last* bin of that interval (last 1250 ms). Total dwell time was aggregated by summing fixation duration over the entire 5-s interval, and thus also included late fixations within a trial. To gain a better understanding of attentional selection early within trial, in each trial we analyzed the onset latency of two fixations: the first fixation on the colored annulus (relevant CS), and the first fixation on the diamond shape (irrelevant distracter). Two participants never looked at the diamond distracter during conditioning and were dropped from this analysis. As can be seen in **Figure 5B** latencies ranged from about 475 to 590 ms, and exhibited a reverse pattern for fixations on the CS (annulus) and the distracter (diamond). An ANOVA with factors CS (L, P, H) and target (CS, distracter) revealed no main effects (both *F* < 1) but a significant interaction, *F*(2,56) = 6.008, *p* = 0.004, $\eta_{p}^{2}$ = 0.177. For both measures, **Table 2** shows significant linear contrasts coding for the influence of shock expectancy. The latency of first fixations on the colored annulus (relevant CS), linearly decreased with increasing shock expectancy, i.e., the stronger the CS-shock association the faster participants were to select and fixate on the CS. In contrast, for first fixations on the diamond distracter, latencies linearly increased. The stronger the shock association of the relevant CS the later participants fixated on the irrelevant distracter within trial. Taken together, in contrast to pupil size and total dwell time, first fixation latencies as a measure of early processing within trial seemed to be exclusively linked to the strength of the shock association.

#### Frequency of Capture by Irrelevant Shape

Although the shape distracter was irrelevant for shock prediction during conditioning, it captured attention because of its bottom-up salience. It was the most salient item of the search display in terms of local feature contrast, because it differed from the two adjacent gray annuli with respect to color *and* shape. **Figure 5C** depicts the frequency of trials in which our participants’ first fixation was on the colored diamond distracter instead of the colored annulus that was the relevant cue for predicting the shock. Capture frequency linearly decreased from about 35% for low expectancy cues to about 22% for high expectancy cues. A one-way ANOVA revealed a significant effect of the CS on capture frequency, *F*(2,62) = 5.237, *p* = 0.008, $\eta_{p}^{2}$ = 0.144. As shown in **Table 2**, a linear contrast coding for associative strength L < P < H was highly significant and was in better accord with the data than a contrast coding for uncertainty or a combined influence of both values. Early processing of the cue’s value, when both, the cue and the distracter, were still represented in the visual periphery thus was strongly linked to the strength of the cue’s association with electric shock. In contrast, the uncertainty of the cue did not influence this early competition between peripheral stimuli.

### Search Task

Our analysis of the dependent variables from the conditioning stage indicated that participants had learned the shock contingencies and acquired associations to encode the differential values of the three color cues when they entered the search task. However, the colors that were previously relevant for predicting shock during conditioning now were task-irrelevant distracters. In contrast, the diamond shape, previously irrelevant for predicting shock during conditioning, now was task-relevant and had to be found and fixated on as quickly as possible. When the search display appeared, the color singleton distracter and the shape singleton target competed for selection as the target of the first fixation.

#### Capture Frequency

**FIGURE 6:**
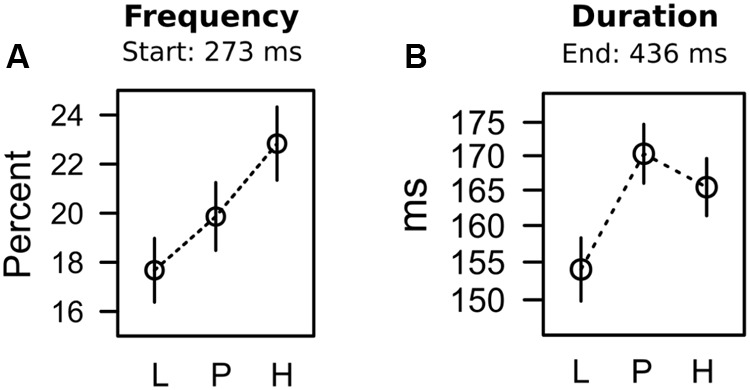
**Frequency (A)** and duration **(B)** of oculomotor capture in the search task.

For each trial, we registered whether the first fixation was on the shape target or on the valued color distracter. Capture frequency was computed as the frequency of trials in which participants fixated the color distracter first. This capture frequency was computed for each of the three different color distracters in each of five successive blocks of search trials. A 3 × 5 ANOVA with factors distracter and block, revealed significant main effects of distracter, *F*(2,62) = 5.050, *p* = 0.009, $\eta_{p}^{2}$ = 0.140, and block, *F*(4,124) = 55.346, *p* < 0.001, $\eta_{p}^{2}$ = 0.331, with no interaction, *F* < 1. The main effect of block existed because capture frequency was at about 25% for all distracters in the first block of the search task, but dropped to about 15% for all distracters in the last block of search trials (*SE* = 2.555%). **Figure 6A** depicts the main effect of distracter color. Again, we used contrast analysis to test whether the observed pattern of means was linked to expectancy, uncertainty, or their combined influence. As shown in **Table 2**, the contrast coding for the linear increase in expectancy L < P < H best fitted with the pattern of data (for pairwise comparisons see **Table 3**). Taken together the statistical results strongly indicated that differences in capture frequency between colors were linked to the different strengths of color-shock associations.

#### Capture Duration

The search task required a fixation on the shape target in order to identify the vertical or horizontal line within the diamond shape that instructed a left-click or right-click with the computer mouse. If participants first fixated on the valued color distracter in capture trials, they had to move their eyes to the shape target eventually to identify the line orientation. We analyzed capture duration as the dwell time on the distracter in capture trials, before participants moved their eyes to the shape target and emitted their manual response. Because capture duration could only be computed if capture actually occurred (on about 20% of the trials as shown in **Figure 6A**), our observations of capture duration were imbalanced, with a varying number of observations per participant, block, and distracter. To account for the imbalanced nature of the data, we fitted a linear mixed model to the unaggregated (per trial) fixation durations, allowing for random variation within participant. An ANOVA estimating the contribution of the fixed distracter term, yielded a significant effect of distracter value, *F*(2,24) = 4.003, *p* = 0.031, $\eta_{p}^{2}$ = 0.249, and contrasts in **Table 2** indicate that the effect was in accord with the combined influence of both expectancy and uncertainty (see **Table 3** for pairwise comparison). The statistical results suggest that differences in capture duration between colors were linked to both, the different strength of color-shock associations, as well as the uncertainty of that prediction (prediction error) during conditioning.

## Discussion

Our results provide further evidence for the hypothesis that fear conditioning changes attention to the CS. The value acquired by the CS did not only affect attentional allocation during conditioning, but moreover, persisted into a subsequent test stage during which shocks were no longer administered and cues previously relevant for predicting shock were introduced as task irrelevant distracters that captured attention automatically. In extension of previous findings (Wang et al., 2013; Schmidt et al., 2015), our results reveal that overt attention is not only influenced by the strength of the shock association but also by the uncertainty of the shock prediction.

### Attention to CS during Fear Conditioning

During fear conditioning, early processing of stimuli appearing in the visual periphery was exclusively linked to the strength of their association with shock. When the stimulus display appeared in conditioning trials, it contained two salient stimuli competing for selection: A colored annulus that was the relevant CS for predicting shock and a colored diamond with high bottom-up salience but no relevance for the prediction of shock. Our results revealed that the CS’s association with shock prevented attentional capture by the irrelevant shape singleton: The stronger the CS-shock association the smaller the probability that the irrelevant diamond shape captured the first fixation (L > P > H). The strength of the shock association thus influenced early attentional selection with all stimuli still being represented in the visual periphery. In contrast, the selection for the first fixation was not affected by the uncertainty of the cues. The same pattern emerged for the latency of the first fixation: The stronger the shock association of the colored annulus, the earlier the onset latency of the first fixation on the annulus (L > P > H) and the later the latency of the first fixation on the diamond distracter (L < P < H). In accord with an evolutionary perspective on fear learning, our results support the idea of a fast and efficient detection of threat-associated stimuli that may provide great adaptive value and improve chances of survival (Ledoux, 1996).

While attentional capture by the color CS was exclusively influenced by the strength of its association with shock, the potential of the CS to *hold* attention seemed to be influenced by uncertainty as well. The total dwell time on the relevant CS did not differ between CSs followed by shock on 100 or 50% of the trials, but exceeded the dwell time on cues never followed by shock (L < P = H). This pattern was not caused by a ceiling effect, but rather was in accord with a combined influence of both, the expectancy (strength of the CS-shock association) and the uncertainty (anticipated prediction error) of the impending shock. From this perspective, the potential of the 50% cue to hold gaze to the same extent as the 100% cue was caused by the combined influenced of a lower shock expectancy but higher uncertainty. One previous experiment has reported that total dwell time was *exclusively* linked to uncertainty during learning about an aversive outcome (Hogarth et al., 2008), with longer fixations on uncertain cues for a loud noise than on predictive cues. For appetitive learning, Koenig et al. (unpublished) reported a similar pattern, with longer fixations on uncertain cues of monetary reward than on predictive cues. The reason we did not observe a pattern L < P > H in the current experiment might be twofold: Firstly, electric shock might provide a biologically stronger US than either a loud noise (Hogarth et al., 2008) or the payment of 10 cent (Koenig et al., unpublished). In turn, association with this more powerful US might have had more influence on fixation dwell time. Secondly, the relative significance of uncertainty in the former two experiments might have been pronounced by the fact that in both experiments participants had to make an explicit prediction of the outcome (Hogarth et al., 2008) or had to perform a predicted motor response (Koenig et al., unpublished). Uncertainty might have been more influential within these designs, because participants were required to indicate their prediction in every trial. Longer dwell time on uncertain cues thus could have been caused by the longer decision time in the face of uncertainty that has indeed been reported for both experiments. In contrast, no explicit prediction of shock was required while participants “passively” observed the stimulus display during aversive conditioning in the current experiment. Taken together, both aspects might have strengthened the relative influence of the US association and created a combined influence of both shock expectancy and shock uncertainty on total dwell time.

A combined influence of expectancy and uncertainty was in accord with the pupil data as well. Here too, the 100% cue and the 50% cue elicited stronger pupil dilation than the 0% cue, but did not significantly differ from one another. This result is in accord with previous studies that reported the pupil’s sensitivity to the coding of both, the strength of a shock association (Reinhard and Lachnit, 2002; Reinhard et al., 2006) as well as uncertainty and error processing in learning and decision making (Satterthwaite et al., 2007; Jepma and Nieuwenhuis, 2011; Preuschoff et al., 2011; Nassar et al., 2012; O’Reilly et al., 2013). A link between pupil dilation and attention has been suggested from different perspectives. Early on, Sokolov (1963) described the pupil response as one component of an integrated orienting response to heighten visual sensitivity, and Wang et al. (2012) recently demonstrated a link of pupil dilation to activity in the superior colliculus as an important physiological substrate of selective visual attention. Lastly, the pupil’s link to the locus coeruleus-norepinephrine (LC-NE) system suggests yet another, neuro-modulatory link to attentional processing (Aston-Jones and Cohen, 2005).

**FIGURE 7:**
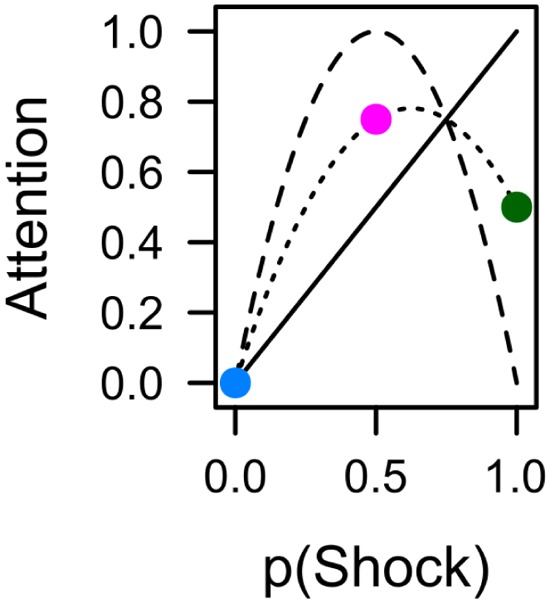
**The average (dotted line) of shock expectancy (solid line) and shock uncertainty (dashed line) yields a simple integration of both values that is in accord with pupil dilation (**Figure 4**) and total dwell time (**Figure 5A**) during conditioning, as well as capture duration in the subsequent search task (**Figure 6B**).** Colors indicate contingencies of 0% (blue), 50% (red), and 100% (green) from the current experiment.

In sum, during fear conditioning, some measures were exclusively linked to the strength of the cues’ shock association (capture frequency, fixation latency) while others (pupil size, total dwell time) seemed to be influenced by an integration of both shock expectancy and shock uncertainty as illustrated in **Figure 7**. One possible explanation of this dissociation derives from the fact that capture frequency and latency were driven by the cues’ representation in the visual periphery, while dwell time and pupil size were influenced by the foveal processing of the cues. However, this dissociation between peripheral and foveal processing also encompasses a temporal aspect. While peripheral processing of the relevant CS was approximately limited to the first 500 ms after stimulus onset, the foveal representation of the CS potentially extended from the time of the first fixation until stimulus offset several seconds later. From this perspective the observed dissociation may well be caused by differences in early versus later processing of the CS (see also Lachnit et al., 2013). As discussed in the next section, such a distinction also fits with the dissociation of capture frequency and capture duration observed in the search task after aversive conditioning.

### Attention to Valued Distracters after Conditioning

Despite their irrelevance in the search task, the CS colors kept drawing attention as a function of their previously acquired shock associations. The frequency of trials in which the valued distracters captured the first fixation, linearly increased with their associative strength (L < P < H). This selection of the CS was “automatic” to the extent that any attention to the color distracter was neither necessary nor in any way strategic for performing the search task. The results are in line with previous experiments demonstrating automatic attentional capture by shock-associated distracter stimuli during extinction (Wang et al., 2013; Schmidt et al., 2015). The intermediate capture rate for the uncertain cue indicates that in accord with associative theory (e.g., Rescorla and Wagner, 1972) and neuro-economic theory (e.g., Schultz et al., 2008) learners computed a value that matched the shock’s probability during conditioning.

If the participant’s first fixation was on the valued distracter in capture trials, capture duration was influenced by the value of the distracter as well. Although colors were irrelevant in the search task, and shocks were no longer administered, the previously shock-associated cues delayed the attentional disengagement from the color distracter necessary to move the eyes to the shape target and previously shock-associated distracters were fixated longer than the distracter never followed by shock during conditioning. Again, the pattern best fitted with a combined influence of both, expectancy and uncertainty, as shown in **Figure 7**.

There is, however, an alternative interpretation of the prolonged capture duration for the uncertain cue that seems plausible at first glance, but must be refuted for several reasons: One could argue that longer dwell time on the partially reinforced distracter during unreinforced search trials was caused by a higher residual shock expectancy of this cue during extinction (and not influenced by uncertainty). Such an effect is referred to as the partial reinforcement extinction effect (PREE) and originally described the finding that an instrumental response is more resistant to extinction after partial reinforcement (Jenkins and Stanley, 1950; Nevin, 1988; Haselgrove et al., 2004). For our Pavlovian fear conditioning procedure one thus might assume that during extinction the shock-association of the partially reinforced cues at some point superseded the shock association of the continuously reinforced cue because of a slower extinction rate of the former and faster extinction of the latter. There are, however, several objections to this reasoning: (1) A PREE is contradicted by our data. Capture frequency, as a measure of expectancy, indicated that the rank order of CS-US associations L < P < H acquired during conditioning was preserved during search. (2) The PREE typically is a between-subject effect. It has been demonstrated that the PREE is limited to between-subject designs (where the 50% vs. 100% condition are different groups of subjects) and vanishes or even reverses for within-subject designs where all cues are presented in the same context (Pearce et al., 1997; Bouton and Sunsay, 2001). (3) The PREE is not established for human Pavlovian fear conditioning. As recently argued by Grady et al. (2016), there is only limited evidence that partial reinforcement in human Pavlovian fear conditioning truly slows the extinction rate.

Having precluded the PREE as a likely explanation for capture duration, we would like to argue that in accord with the dissociation between attentional measures in the conditioning stage (see our discussion above) the search task exhibited the same dissociation, where capture frequency was driven by shock expectancy while capture duration was linked to both, shock expectancy and uncertainty. Again this dissociation was related to early processing of the distracter in the visual periphery (capture frequency) versus later processing of the distracter represented at the fovea. As stated in **Figure 6**, capture frequency was determined by the oculomotor decision to select the distracter for the first fixation, and these distracter fixations started around 273 ms. In contrast, capture duration was driven by the attentional disengagement from the distracter, where distracter fixations ended around 436 ms.

The current experiment does not allow to conclude whether the dissociation between measures was linked to their temporal dissociation (early vs. late) or spatial dissociation (peripheral vs. foveal). However, it seems unlikely that learners – given enough time – should not be able to decode the uncertainty of a peripheral shock-associated color cue in principle. On the other hand, an earlier representation of shock association again makes sense from an evolutionary perspective. If a fast and efficient detection of threat-associated stimuli is crucial for survival (Ledoux, 1996), the probability of an impending threat must be represented fast and efficiently, whereas the uncertainty of this probabilistic prediction is subordinate at first, but is also retrieved with some delay. A sequential processing of expectancy and uncertainty has also been reported for the brains dopamine system (Fiorillo et al., 2003; Schultz et al., 2008), which has been shown to comply with basic predictions of associative learning theories (Waelti et al., 2001) and is also linked to the motivational salience of reward-associated stimuli (Hickey and Peelen, 2015) and attentional capture (Anderson et al., 2016). In particular, the response of dopamine neurons has been described as biphasic with a first fast component that linearly increases with the probability of reward and a second slow component that gradually builds up to represent reward uncertainty (Fiorillo et al., 2003). The involvement of dopamine signaling in aversive conditioning, however, is less well understood (Schultz, 2016). As outlined in the introduction, physiological evidence from human fear conditioning (e.g., Dunsmoor et al., 2007) also suggests that both values are represented in the brain, where different regions are involved in the processing of expectancy (amygdala, anterior cingulate) and uncertainty (insula, dorsolateral prefrontal cortex).

The observation that capture duration was not exclusively linked to uncertainty deviated from the results of Koenig et al. (unpublished) who reported that uncertain cues of high reward exceeding both, reliable cues for high reward and reliable cues for low reward. As discussed above for the total dwell time during conditioning, this deviation could have been caused by a relatively stronger representation of shock expectancy (or weaker representation of uncertainty). To repeat our previous point, pairings with electric shock might indeed result in a stronger value than pairings with monetary gains. In line with this possibility, Wang et al. (2013) found that a non-salient distracter acquired the potential to elicit attentional capture after pairings with monetary loss or electric shock as in the current experiment, but not after pairings with monetary gains as used by Koenig et al. (unpublished).

### Theoretical Implications and Conclusion

In sum, our findings provide clear evidence that fear conditioning creates an automatic attention bias for the CS that depends on their correlation with the aversive outcome. This bias was exclusively linked to the strength of the cues’ shock association for the early attentional processing of cues in the visual periphery (capture frequency), but additionally was influenced by the uncertainty of the shock prediction after participants fixated on the cues (capture duration).

An attentional bias according to the strength of an association with reward has been previously suggested by Gottlieb (2012) as an “attention for liking” mechanism. However, attentional capture by shock-associated stimuli clearly cannot be subsumed under a mechanism that promotes *reward*-seeking behavior. Le Pelley et al. (2016) recently suggested a model in which the attentional bias weight for a cue is driven toward its associative strength to cause “attention to high expectancy cues.” In the tradition of formal learning theories, this association may be an association with either reward or punishment. The current results also suggest that both, expectancy *and* uncertainty, may be represented sequentially within trial, and that a bias for uncertain cues selectively affects measures of attentional holding (total dwell time, capture duration). This latter aspect is in accord with the attentional learning theory of Pearce and Hall (1980; Pearce et al., 1982) in which the attentional bias weight of a cue is driven toward its absolute prediction error to cause “attention to uncertain cues.”

Our results do not speak to the essential assumption of attentional learning theories that the link between learning and attention actually is bidirectional. Not only should learning affect attention, but also should attention influence future learning, with faster learning for attended cues. Accordingly, the “attentional” learning theories of Mackintosh (1975) and Pearce and Hall (1980) originally stated that learning should alter a cue’s *associability*, and it is only a secondary assumption that this alteration is by means of attentional processes. An important direction for future research from this perspective thus would be to establish a causal link between measures of attention and the learning rate (associability) during associative learning. For example, with respect to the current findings, it remains to be shown whether learning would be affected by the earlier or more frequent selection of a cue within trial (attention to high expectancy cues), or whether learning would rather be influenced by the duration of fixations on the cue (attention to high uncertainty cues).

Another topic that we did not address in the current experiment is how attentional capture after fear conditioning was related to the participants’ explicit awareness of the associations between colors and electric shock. We did not include contingency ratings during or after the conditioning stage because they could have created contingency awareness that would not have existed without those ratings. Also, contingency ratings at the end of the experiment would have been affected by extinction learning since shocks were not administered during search. At any rate, the correspondence between contingency awareness and attentional capture remains an open question for future research.

## Author Contributions

SK is primarily responsible for the design, execution, and analysis of the experiment. MU and HL have made significant contributions to the conception of the experiment and revisions of the manuscript.

## Conflict of Interest Statement

The authors declare that the research was conducted in the absence of any commercial or financial relationships that could be construed as a potential conflict of interest.

## Footnotes

1 http://www.neurobs.com
